# High-Pressure Oxidation of Ammonia Mixed with Dimethoxymethane

**DOI:** 10.1021/acs.energyfuels.5c01020

**Published:** 2025-07-09

**Authors:** Katiuska Alexandrino, Álvaro Andrés, Alicia Callejas, María U. Alzueta

**Affiliations:** Aragón Institute of Engineering Research (I3A), Department of Chemical and Environmental Engineering, 16765 University of Zaragoza, Zaragoza 50018, Spain

## Abstract

The oxidation of ammonia-dimethoxymethane (NH_3_-DMM) mixtures at high pressure was analyzed from both experimental and kinetic modeling points of view. Experiments were performed using a laboratory tubular flow reactor installation and were conducted at 10, 20, and 40 bar under fuel-rich (λ = 0.7), stoichiometric (λ = 1), and fuel-lean (λ = 3) conditions and temperatures ranging from 650 to 1250 K. The inlet DMM concentration was varied (100 and 200 ppm), keeping the concentration of ammonia constant at 1000 ppm. The data were interpreted in terms of a detailed chemical kinetic model. Despite some discrepancy between the model predictions and measurements, the model accurately followed the experimental trends, highlighting its ability to describe the oxidation of the NH_3_-DMM mixture. The experimental and predicted results suggested that the conversion of both ammonia and DMM was favored by increased pressure and higher inlet concentrations of O_2_ and DMM. Under the conditions of the present work, the dominant path for ammonia and DMM conversion leads to N_2_/N_2_O and CO/CO_2_ in any case. Concentrations of NO and NO_2_ were below the detection limit in all the experimental conditions studied, which imply a benefit in the reduction of NO_*x*_ emissions during the combustion of pure ammonia. DMM enhanced the ammonia reactivity, although its presence leads to the formation of CO_2_ not only by the common CO + OH reaction but also through the interaction of N_2_O with CO.

## Graphical abstract

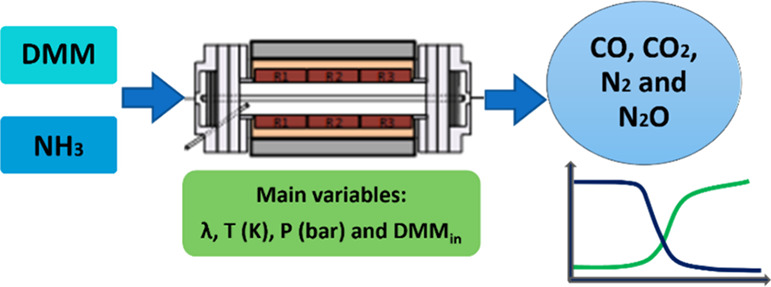

## 1 Introduction

One way to mitigate the CO_2_ emission from the burning fossil fuels, which is one of the main culprits of global warming, is to use alternative fuels such as biorenewable or non-carbon-based fuels. Ammonia (NH_3_) has received increasing attention as a hydrogen carrier and carbon-free fuel to be used in different combustion processes, such as internal combustion engines, and has been proposed to be a potential future fuel.^1^ Some of its advantages are the high-octane rating of 110–130,^2^ the relatively high power-to-fuel-to-power (PFP) efficiency,^3^ and the fact that its production, storage, handling, and distribution facilities are available worldwide.^4^ As a potential future fuel, a detailed understanding of ammonia oxidation processes has been of great interest, and different experimental and modeling studies on its chemical conversion have been reported in the literature^5–9^ However, ammonia also has several barriers to overcome before it can be used as a fuel, such as the significant levels of NO_*x*_ emissions during combustion, mainly NO, and difficulties in autoignition (low reactivity),^10^ which are the main barriers for ammonia use in combustion devices.

To deal with the low ammonia reactivity, the use of mixtures of ammonia with highly reactive fuels has been proposed. A detailed understanding of the oxidation processes of this dual-fuel approach is crucial for the use of ammonia as a potential future fuel. In this sense, studies addressing the co-burning of ammonia with hydrogen (H_2_), methane (CH_4_), syngas, and different oxygenated compounds such as methanol (CH_3_OH), ethanol (C_2_H_5_OH), dimethyl ether (DME, C_2_H_6_O), diethyl ether (DEE, C_4_H_10_O), and dimethoxymethane (DMM, C_3_H_8_O_2_) have shown that these ignition promoters play a crucial role in tailoring the autoignition behavior of ammonia for its applicability in engines and turbines^11–21^

The mixing of ammonia and biomass-oxygenated fuels could be a promising possible carbon-neutral fuel, and among the different alternatives, oxymethylene dimethyl ethers (OMEs, CH_3_O-(CH_2_O)_*n*_-CH_3_, *n* = 1–5), which are oligomers of the formaldehyde monomer (O=CH_2_), have attracted considerable interest due to different reasons such as their nontoxicity, high cetane number, high oxygen content, and no C–C bonds resulting in a low ability to form soot.^22^ Dimethyl ether (DME, CH_3_O-(CH_2_O)_*n*_-CH_3_, *n* = 0; OME0) is the most simple OME. NH_3_-DME mixtures have already been tested in CI engines,^4^ and their chemical conversion has also been studied at the laboratory scale,^15,23–25^ showing DME to be effective in promoting the ammonia reactivity. Dimethoxymethane (DMM, CH_3_O-(CH_2_O)_*n*_-CH_3_, *n* = 1; OME1) is another important OME, which has a lower vapor pressure and higher oxygen content compared to DME.^26^ DMM can be produced in a sustainable carbon cycle to achieve zero-net CO_2_ emission.^27^ During the production of OMEs, the biomass, mainly lignocellulose from crop residues, energy crops, and forest and municipal wastes, is converted into methanol through a gasification process. Subsequently, methanol reacts with formaldehyde to form DMM through a heterogeneously catalyzed reactive distillation.^28^ The DMM chemical conversion has been widely studied in the literature,^26,29–31^ and its very low capacity to form soot under pyrolytic conditions has also been demonstrated.^22^

Despite DMM being a promising additive, few studies on NH_3_-DMM mixture combustion behavior are available in the literature. Elbaz et al.^18^ measured the laminar burning velocity for various NH_3_-DMM mixtures (DMM mole fraction varied from 0.2 to 0.6) in a constant-volume spherical vessel. The measurements were performed at 298 K, 0.1 MPa, and equivalence ratios over the range of 0.8–1.3. The results showed that the addition of DMM enhanced the combustion characteristics of ammonia. Also, a chemical kinetic model of NH_3_-DMM, aimed at interpreting the high-temperature combustion chemistry of NH_3_-DMM mixtures, was able to capture reasonably well the experimental data. Reaction path analyses indicated that the oxidation of the NH_3_-DMM mixture can be treated as a parallel oxidation process of individual fuels, which compete for the same radical pool (OH, H, and O), thus making the chemical system interwoven. Dai et al.^17^ measured the ignition delay times (IDTs) of NH_3_-DMM mixtures at DMM blending ratios of 5, 10, 15, and 50%, pressures of 1 and 10 bar, equivalence ratio of 0.5, and temperature range of 1193–1852 K. A detailed NH_3_-DMM model was proposed with the addition of the cross-reactions between DMM and ammonia, i.e., prompt NO and reburn reactions, recombination reactions, H-abstraction reactions, and disproportionation reactions. That model predicted well the IDTs measured in that study. Zhang et al.^32^ carried out a numerical study on the combustion properties of NH_3_-DME and NH_3_-DMM mixtures by constructing a chemical mechanism and tested its accuracy using measured laminar burning velocities and ignition delay times of both NH_3_-DMM and NH_3_-DME mixtures from the literature.

To the best of our knowledge, studies addressing the species profiles during the combustion of NH_3_-DMM mixtures, which are vital for validating chemical kinetic models, are still lacking in the literature. Moreover, most of the available data are limited to low pressures or narrow operating conditions, which may not represent the range relevant to practical combustion systems. Therefore, the present work aims to fill this gap by performing high-pressure experiments with NH_3_-DMM mixtures in a flow reactor under a broad range of operating conditions. Specifically, measurements of the reactants and main products resulting from the chemical conversion were carried out by varying the temperature (650–1250 K), pressure (10, 20, and 40 bar), stoichiometry (λ = 0.7, 1, and 3), and inlet DMM concentration (100 and 200 ppm).

To gain further insight into the chemical conversion of the NH_3_-DMM system, a chemical kinetic model was compiled from the literature and validated against both the experimental results obtained in this work and experimental IDT data from the literature. This study contributes to a deeper understanding of ammonia oxidation when blended with a highly reactive fuel such as DMM, particularly under extended operating conditions, thereby supporting the advancement of dual-fuel combustion systems for low-carbon mobility applications.

## 2 Experimental Methodology

### 2.1 Experimental Setup

Figure 1 presents a schematic of the experimental setup used to carry out the oxidation experiments of the NH_3_-DMM system at high pressure. This installation has been successfully employed by our research group and has been described in detail in previous works.^9,33^ Therefore, only a brief overview is provided here.

**Figure 1:**
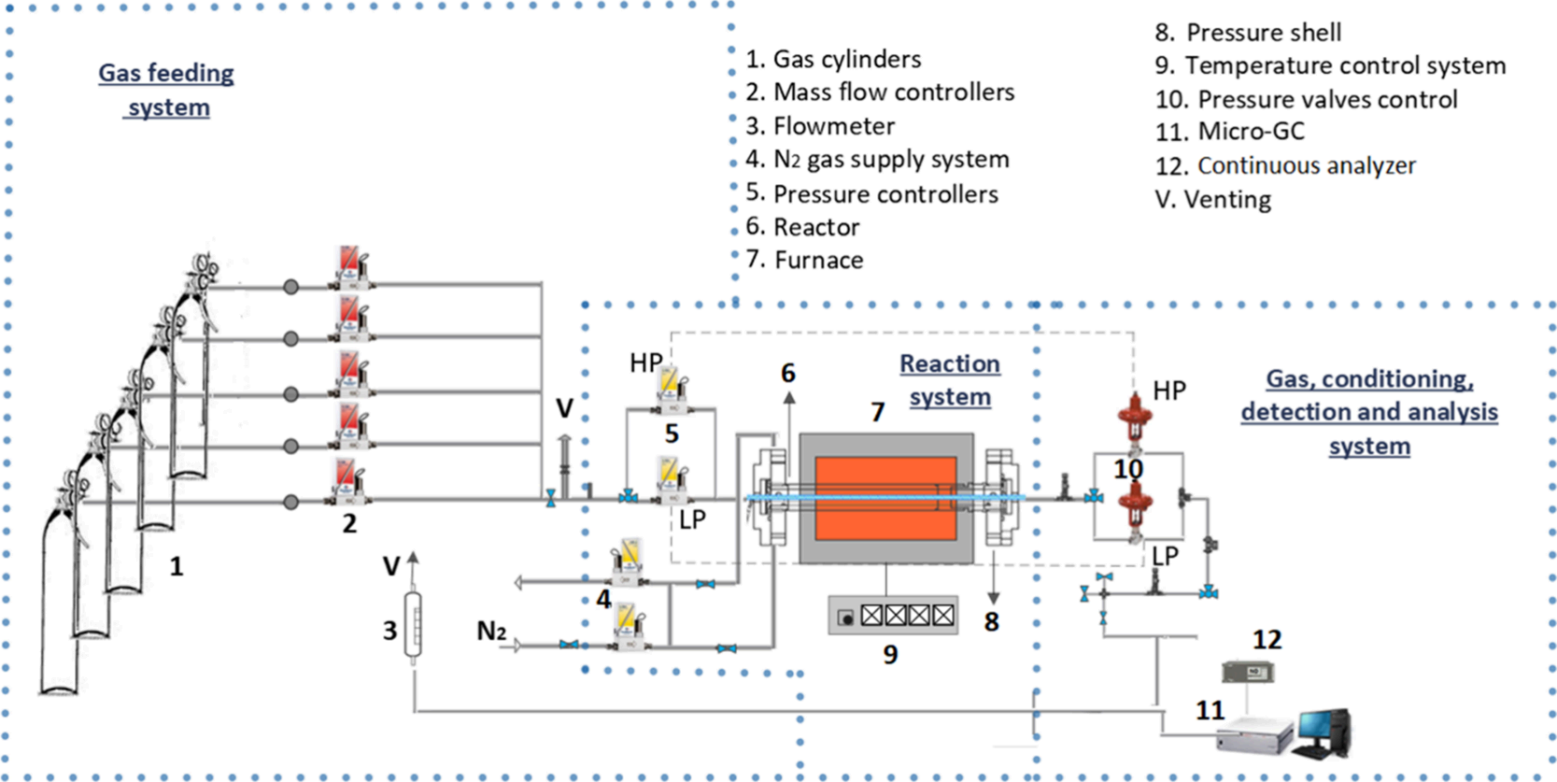
Experimental setup for experiments at high pressure.

The reactant gases (NH_3_, DMM, and O_2_ diluted in argon), with Ar to balance, are fed to the reactor from gas cylinders (Air Liquide, Praxair, or Messer) using calibrated mass flow controllers and mixed before entering the quartz flow reactor of 1538 cm length and 6 mm inner diameter.

The quartz flow reactor is enclosed in a stainless-steel tube that acts as a pressure shell, which in turn is placed horizontally in a three-zone electrically heated furnace with individual temperature control ensuring an isothermal reaction zone of approximately 500 mm. The isothermal region was identified experimentally by obtaining longitudinal temperature profiles under various temperature and pressure conditions in an Ar atmosphere (1000 mL (STP)/min, the same flow rate for all experimental runs). The temperature measurements were carried out using a thermocouple placed between the quartz tube and the steel shell. As an example, Figure S1 displays the temperature profiles obtained at 40 bar for different temperatures.

The system is pressurized from the feed gas cylinders, and the pressure inside the reactor is monitored upstream of the reactor by a differential pressure transducer and controlled by a pneumatic pressure valve situated after the reactor. The pressure in the shell-side of the reactor is kept close to that inside the reactor to avoid sudden pressure gradients and to prevent rupture of the quartz tube.

Downstream the reactor, the system pressure is reduced to atmospheric pressure before the product gas stream passes through a condenser to be conditioned for further analysis in a micro gas chromatograph (micro-GC) (Agilent 3000A) equipped with TCD detectors (used to quantify CO and CO_2_) and in an ABB continuous analyzer (model Advance Optima AO2020) for NH_3_, NO, NO_2_, and N_2_O, which provides real-time, highly accurate measurements of gas concentrations across a range of 0–2000 ppm. The estimated uncertainty of the measurements is within ±5% but not less than 5 ppm for the continuous analyzer and 10 ppm for the micro-GC.^9^

Table 1 lists the experimental conditions used. The amount of O_2_ necessary to perform each oxidation experiment has been calculated through the air excess ratio (λ), defined as the ratio between the inlet oxygen and the stoichiometric oxygen, considering the stoichiometry of both reactions: NH_3_ + 0.75O_2_ ⇄ 0.5N_2_ + 1.5H_2_O and C_3_H_8_O_2_ + 4O_2_ ⇄ 3CO_2_ + 4H_2_O (eq 1) (λ = 1 means stoichiometric conditions; λ < 1 means fuel-rich conditions; λ > 1 means fuel-lean conditions). Argon has been used to balance the total flow rate up to 1000 mL (STP)/min (highly diluted conditions to minimize thermal effects due to reaction), which gives a temperature- and pressure-dependent gas residence time in the isothermal reaction zone, as given by eq 2.

**Table 1:** Experimental Conditions^a^

set	NH_3_ (ppm)	DMM (ppm)	O_2_ (ppm)	λ	*P* (atm)
1	1011	112	863	0.72	10
2	1034	127	1194	0.93	10
3	1015	131	3586	2.79	10
4	1034	110	856	0.70	20
5	1001	129	1233	0.97	20
6	976	123	3482	2.84	20
7	988	132	815	0.64	40
8	980	110	1213	1.03	40
9	982	126	3464	2.80	40
9R	977	117	3438	2.86	40
10	1004	228	1564	0.94	20
11	916	220	1496	0.95	40
12	903	213	4582	3.0	40

a Ar is used to balance the total flow rate up to 1000 mL (STP)/min.

$$\lambda =\frac{O_{2\;\mathrm{inlet}}}{0.75\times {{\mathrm{NH}}_{3\;}}_{\mathrm{inlet}}+4\times C_{3}H_{8}{O_{2\;}}_{\mathrm{inlet}}}$$ 1

$$t_{r}(s)=231.6\frac{P(\mathrm{bar})}{T(K)}$$ 2

Using Ar as bath gas allows the quantification of N_2_ formed from ammonia and therefore the determination of the N balance. Sets 9 and 9R are repeated experiments that allow one to evaluate the repeatability of the experiments.

The experiments have been carried out in the temperature range of 650–1250 K, using approximately 1000 ppm of ammonia, in which the influence of pressure (10, 20, and 40 bar), λ (approximately 0.7, 1, and 3), and inlet DMM concentration (around 100 and 200 ppm) is evaluated.

Sets 1–9 have been used to analyze the influence of pressure and stoichiometry for a mixture of around 1000 ppm of ammonia and 100 ppm of DMM. Also, the influence of pressure has been analyzed for an inlet DMM concentration of 200 ppm and λ = 1 though experimental sets 10 and 11, while sets 11 and 12 have allowed one to evaluate the effect of stoichiometry for and inlet DMM concentration around 200 ppm at 40 bar. Additionally, the influence of the inlet DMM concentration has been analyzed for λ = 1 at 20 bar (sets 5 and 10) and 40 bar (sets 8 and 11) and for λ = 3 at 40 bar (sets 9 and 12).

### 2.2 Chemical Kinetic Model

The experimental results have been compared with modeling predictions using a chemical kinetic model built taking as starting point the model proposed by García-Ruiz et al.^34^ on the high-pressure oxidation of NH_3_-CH_4_ mixtures, which is founded on the nitrogen chemistry of Glarborg et al.^35^ drawing on more recent work on amine chemistry by Stagni et al.,^36^ updated by adding subsets for various species^37,38^ and including modifications and/or recommendations of recent studies.^39–46^ In the present work, that model was extended by including the DMM submechanism used in the work of Marrodán et al.^47^ on the high-pressure oxidation of C_2_H_2_-DMM mixtures, which was originally taken from the work of Marrodán et al.^29^ on the high-pressure oxidation of DMM in a tubular flow reactor and was revised, updated, and modified by Marrodán et al.^47^ These modifications included the addition of new reactions to the DMM submechanism and the change of the kinetic parameters of some reactions according to Vermeire et al.^48^ Moreover, in the present work and to include the C–N interactions, the submechanisms for the recombination reactions between CH_3_OCH_2_OCH_2_/CH_3_OCH_2_O/CH_3_OCH_2_ and NH_2_, H atom abstraction reactions of CH_3_OCH_2_OCH_3_/CH_3_OCHO/CH_4_/CH_3_OH/CH_2_O by NH_2_/NO/NO_2_ radicals, and the disproportionation reactions between CH_3_OCH_2_O/CH_3_O/CH_2_OH/HCO and NO/NH_2_, proposed by Dai et al.,^17^ were included. Furthermore, to better fit the simulation results to the experimental data, the kinetic parameters of few reactions were modified, as indicated in Table 2.

**Table 2:** Modified Reactions^a^

reaction		*A*	*n*	*E*_a_
R1 ^49^	CH_3_OCH_2_OCH_3_ + CH_3_O_2_ ⇄ CH_3_OCH_2_OCH_2_ + CH_3_O_2_H	1.06e^1^	3.557	1.26e^4^
R2 ^49^	CH_3_OCH_2_OCH_3_ + CH_3_O_2_ ⇄ CH_3_OCHOCH_3_ + CH_3_O_2_H	1.81e^2^	3.163	1.17e^4^
R3 ^32^	CH_3_OCH_2_OCH_3_ + NH_2_ ⇄ CH_3_OCH_2_OCH_2_ + NH_3_	1.80	3.610	4353
R4 ^32^	CH_3_OCH_2_OCH_3_ + NH_2_ ⇄ CH_3_OCHOCH_3_ + NH_3_	3.79e^3^	2.426	4475
R5 ^17^	HCO + M ⇄ H + CO + M	5.7e^11^	0.660	1.48e^4^
R6 ^17^	HCO + O_2_ ⇄ CO + HO_2_	7.58e^12^	0.000	4.1e^2^
R7 ^49^	NH_2_ + OH ⇄ NH + H_2_O	4.04e^4^	2.520	–616

a Units: s, cm^3^, cal, mol.

Reactions R1, R2, and R5–R7 were already in the base model, while reactions R3 and R4 were among the reactions added from the work of Dai et al.^17^ The kinetic parameters of the H atom abstraction reactions by CH_3_O_2_ (reactions R1 and R2) were updated with those proposed by Jacobs et al.^49^ and also used by Dai et al.^17^ These rate coefficients were derived based on those for H atom abstraction by HO_2_ radicals. Specifically, Jacobs et al.^49^ decreased by 20% the A-factor of the reactions between DMM and HO_2_ radicals proposed by Vermeire et al.^48^ The kinetic parameters of reactions R3 and R4 used by Dai et al.,^17^ taken from Allen,^50^ were replaced by those proposed by Zhang et al.,^32^ who estimated them based on the NH_3_/DME/air mechanism proposed by Xiao and Li.^24^ The change of the kinetic parameters mainly in reactions R3 and R4 allowed a better fit of the model to the experimental data of DMM, mainly at 20 bar and λ = 3 and all the experiments at 40 bar, where the model initially greatly shifted the DMM concentration profile to lower temperatures, compared to the experimental concentration profile.

Figure 2 shows an example of the comparison of the simulations obtained for λ = 3 at 20 bar and λ = 1 at 40 bar using the model of Dai et al.,^17^ our model with the kinetic parameters of R3 and R4 of the work of Dai et al.,^17^ and our model with the kinetic parameters of R3 and R4 of the work of Zhang et al.^32^

**Figure 2:**
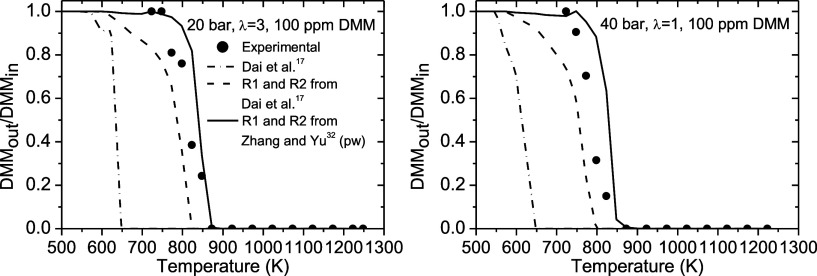
Comparison of the modeling results using the model of Dai et al.,^17^ our model with the kinetic parameters of R3 and R4 of the work of Dai et al.,^17^ and our model with the kinetic parameters of R3 and R4 of the work of Zhang et al.^32^ Sets 6 and 8 are shown in Table 1.

It is evident that the use of the parameters of reactions R3 and R4 from Zhang et al.^32^ shifts the DMM concentration profile further to the right, resulting in a much better fit with the experimental data compared to using the parameters from Dai et al.^17^ Therefore, the final model used in the subsequent simulations adopts the parameters from Zhang et al.^32^ These results indicate that reactions R3 and R4 may play important roles in the oxidation of DMM at high-pressure and fuel-lean conditions and need deeper exploration. The full model compiled in the present work (223 chemical species and 1714 reactions) as well as the thermodynamic data taken from the same sources as for the kinetic mechanisms is provided in the Supporting Information. Numerical calculations have been conducted with the plug-flow reactor module of the CHEMKIN-PRO software package.^51^ The simulations were carried out using the temperature within the isothermal zone, given that the results showed no significant differences when using measured temperature profiles. The final model has been used to simulate not only the data obtained in the present work but also the ignition delay time data measurements of NH_3_-DMM mixtures from the work of Dai et al.,^17^ whose results are shown in Figure S2 in the Supporting Information. The results illustrate that the model, in general, predicts the experimental ignition delay time data.

## 3 Results and Discussion

The influence of the stoichiometry on the oxidation of NH_3_-DMM mixtures has been evaluated at different pressures (10, 20, and 40 bar) for an inlet DMM concentration of around 100 ppm and at 40 bar for a DMM concentration of around 200 ppm. Figure 3 shows, as an example, the experimental results (symbols) and modeling calculations (lines) for the concentrations of NH_3_, N_2_, N_2_O, DMM, CO, and CO_2_, as the main products quantified, obtained at 10 bar and an inlet DMM concentration of 100 ppm. To facilitate comparison of the concentration profiles of NH_3_ and DMM for the different λ values, the concentrations of these reactants have been normalized with respect to their inlet concentrations. Concentration profiles of NO and NO_2_ have not been shown because the concentration of these products was always below the detection limit (5 ppm). This is in line with that observed by Song et al.^6^ in their work on the NH_3_ oxidation at high pressures in a laminar flow reactor, where the products of reaction were found to be N_2_ and N_2_O, while the concentrations of NO and NO_2_ were below the detection limit.

**Figure 3:**
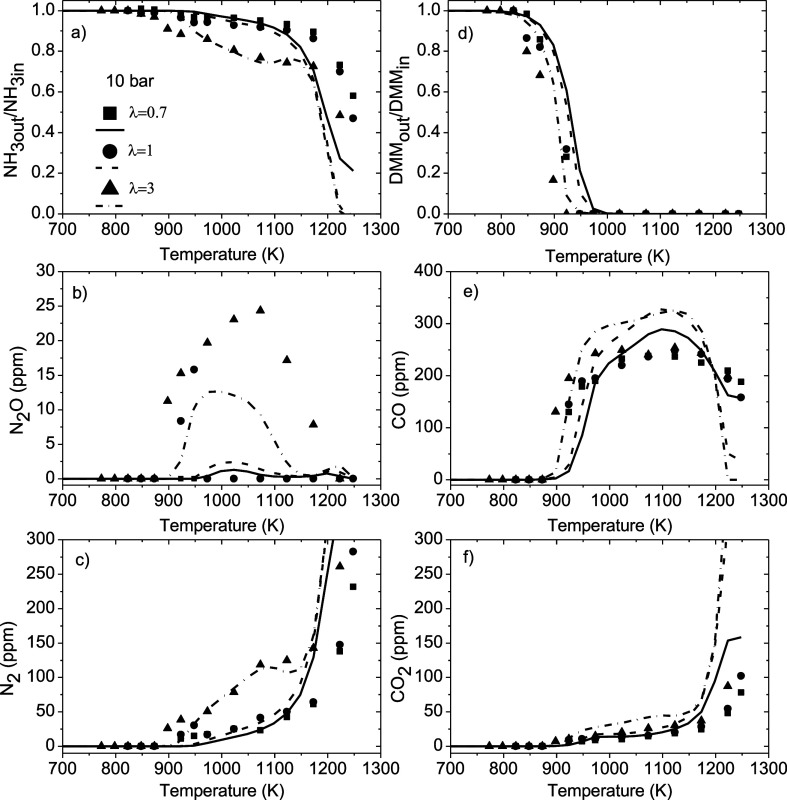
Influence of the stoichiometry on the concentration profiles of (a) NH_3_, (b) N_2_O, (c) N_2_, (d) DMM, (e) CO, and (f) CO_2_ during the NH_3_-DMM mixture oxidation as a function of the temperature at 10 bar and an inlet DMM concentration of 100 ppm. Experimental results are denoted by symbols, and modeling calculations are denoted by lines. Sets 1–3 in Table 1.

The concentration profiles of reactants and products for 100 ppm of DMM at 20 and 40 bar and for 200 ppm of DMM at 40 bar can be seen in Figures S3–S5, respectively, in the Supporting Information. Figure S4 also shows the repeated experiment performed at 40 bar, λ = 3, and inlet DMM concentration of approximately 100 ppm (set 9R in Table 1). The repeatability of the experiments is pretty good, indicating the good performance of the experimental system and procedure.

The modeling predictions follow the experimental trends, although some discrepancy between the model predictions and measurements is observed. The discrepancies could be due to the formation of C–N species, whose exact nature remains unclear, as has been reported by Marrodán et al.^38^ and Alzueta et al.,^52^ and it would be valuable to conduct further study in this regard.

Figures 3 and Figures S3–S5 indicate that, for any pressure and inlet DMM concentration studied, the onset temperature for consumption of both NH_3_ and DMM—and therefore the onset temperature for the formation of products—is shifted to lower temperatures as the amount of oxygen fed to the system increases, being more evident for fuel-lean conditions. The importance of stoichiometry for the conversion of neat NH_3_ and DMM at high pressure has also been reported previously.^9,29^

To delineate the main reaction routes through which the conversion of ammonia and DMM proceeds during the conversion of the NH_3_-DMM mixtures, reaction pathway analyses under the conditions of the experiments were carried out using the proposed model. The main features were similar for all cases, and Figures 4 and 5 show the reaction pathway diagram for ammonia and DMM, respectively.

**Figure 4:**
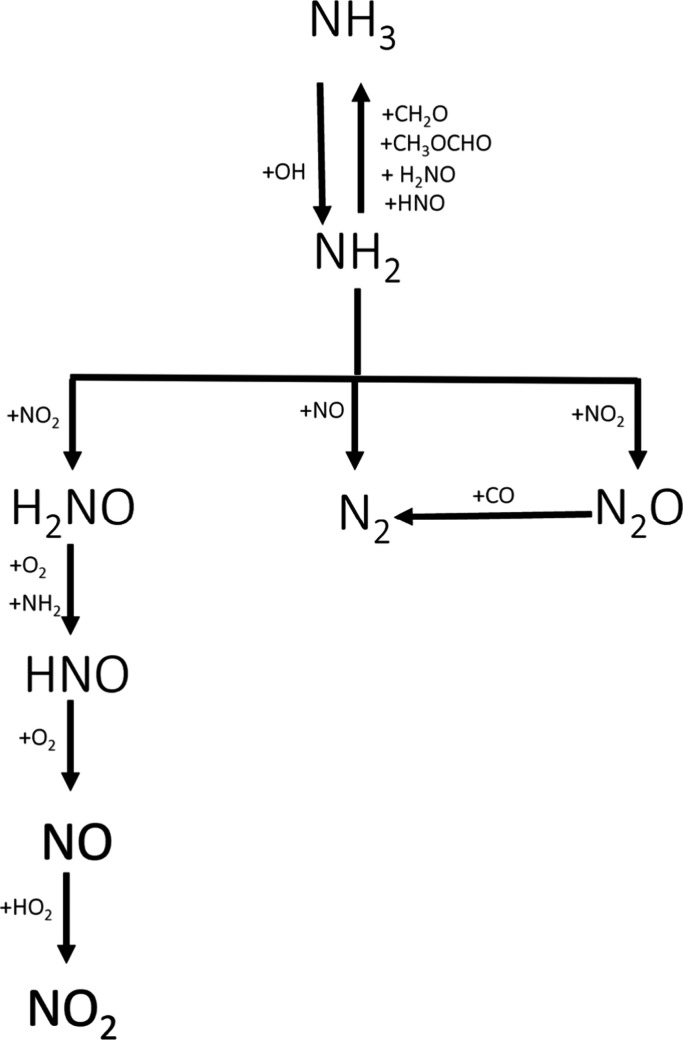
Reaction pathway diagram for the conversion of ammonia during the oxidation of NH_3_-DMM mixtures at a high pressure.

**Figure 5:**
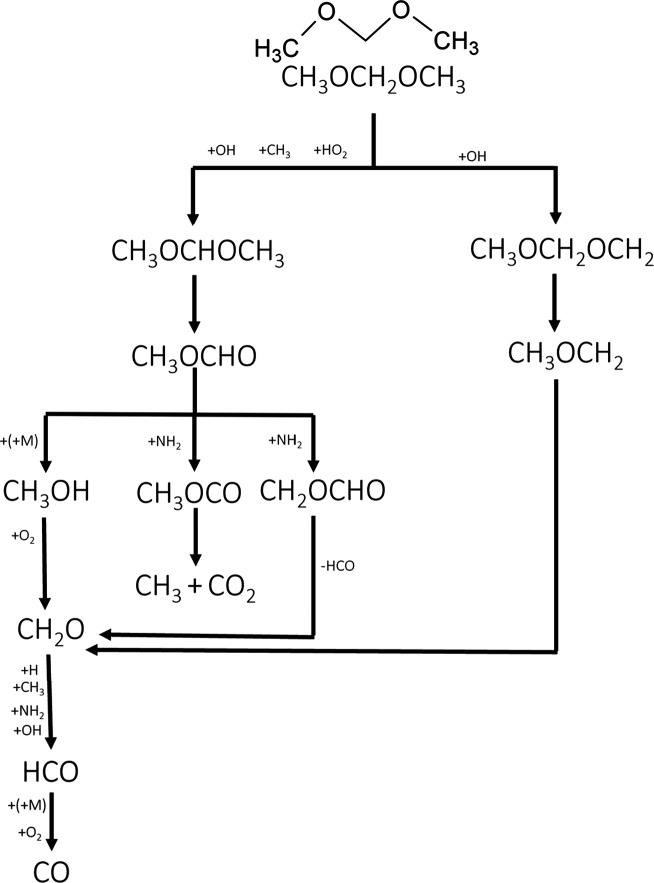
Reaction pathway diagram for the conversion of DMM during the oxidation of NH3-DMM mixtures at high pressure.

NH_3_ is consumed by reaction 3 for all of the experimental conditions studied.

$${\mathrm{NH}}_{3}+\mathrm{OH}⇄{\mathrm{NH}}_{2}+H_{2}O$$ R8

NH_2_ radicals produced react with carbonaceous species, specifically with methyl formate (CH_3_OCHO) and formaldehyde (CH_2_O), to finally produce CO/CO_2_ through reactions 4–9.

$${\mathrm{CH}}_{3}\mathrm{OCHO}+{\mathrm{NH}}_{2}⇄{\mathrm{CH}}_{3}\mathrm{OCO}+{\mathrm{NH}}_{3}$$ R9

$${\mathrm{CH}}_{3}\mathrm{OCO}⇄{\mathrm{CH}}_{3}+{\mathrm{CO}}_{2}$$ R10

$${\mathrm{CH}}_{3}\mathrm{OCHO}+{\mathrm{NH}}_{2}⇄{\mathrm{CH}}_{2}\mathrm{OCHO}+{\mathrm{NH}}_{3}$$ R11

$${\mathrm{CH}}_{2}\mathrm{OCHO}⇄{\mathrm{CH}}_{2}O+\mathrm{HCO}$$ R12

$${\mathrm{CH}}_{2}O+H/{\mathrm{CH}}_{3}/{\mathrm{NH}}_{2}/\mathrm{OH}⇄\mathrm{HCO}+H_{2}/{\mathrm{CH}}_{4}/{\mathrm{NH}}_{3}/H_{2}O$$ R13

$$\mathrm{HCO}+(+M)/O_{2}⇄\mathrm{CO}+(+M)/{\mathrm{HO}}_{2}$$ R14

NH_2_ also reacts with nitrogenous species, specifically with NO and NO_2_, to yield N_2_ and N_2_O, respectively (reactions 10 and 11).

$${\mathrm{NH}}_{2}+\mathrm{NO}⇄N_{2}+H_{2}O$$ R15

$${\mathrm{NH}}_{2}+{\mathrm{NO}}_{2}⇄N_{2}O+H_{2}O$$ R16

The nondetection of NO or NO_2_ in any of the experiments could be because these species are rapidly consumed to give N_2_ and N_2_O through reactions 10 and 11. It is observed through modeling calculations that N_2_O is further oxidized by CO to give N_2_ and CO_2_ (reaction 12).

$$N_{2}O+\mathrm{CO}⇄N_{2}+{\mathrm{CO}}_{2}$$ R17

In this sense, CO_2_ is produced by the common reaction of CO with OH radicals (reaction 13) and by the reaction of N_2_O with CO (reaction 12).

$$\mathrm{CO}+\mathrm{OH}⇄{\mathrm{CO}}_{2}+H$$ R18

DMM is consumed mainly by H-abstraction reactions 14 and 15.

$${\mathrm{CH}}_{3}{\mathrm{OCH}}_{2}{\mathrm{OCH}}_{3}+\mathrm{OH}/{\mathrm{CH}}_{3}/{\mathrm{HO}}_{2}⇄{\mathrm{CH}}_{3}{\mathrm{OCHOCH}}_{3}+H_{2}O/{\mathrm{CH}}_{4}/H_{2}O_{2}$$ R19

$${\mathrm{CH}}_{3}{\mathrm{OCH}}_{2}{\mathrm{OCH}}_{3}+\mathrm{OH}⇄{\mathrm{CH}}_{3}{\mathrm{OCH}}_{2}{\mathrm{OCH}}_{2}+H_{2}O$$ R20

The consumption of ammonia and DMM is favored under fuel-lean conditions because the formation of OH radicals is maximized, promoting the occurrence of the reactions that lead to the consumption of ammonia and DMM.

DMM produces CH_3_OCHOCH_3_ and CH_3_OCH_2_OCH_2_ radicals (Figure 5), which yield methyl formate and CH_3_OCH_2_ radicals, respectively. The last ones decompose to give formaldehyde, which is subsequently oxidized to CO through the pathway CH_2_O → HCO → CO, in agreement with earlier studies by our group.^53^ Methyl formate can also produce formaldehyde or react with NH_2_ radicals to produce CH_3_OCO and CH_2_OCHO radicals through reactions 4 and 6. The CH_3_OCO radicals decompose to produce CH_3_ and CO_2_ (reaction 5), while the CH_2_OCHO radicals decompose to yield formaldehyde and HCO radicals (reaction 7).

To determine if the measured species were dominant under the studied conditions and to evaluate the goodness of the experiments, the atomic N and C balances were determined for all the experiments. Figure 6a,b shows examples of N and C atom balances for different stoichiometries, pressures, and inlet DMM concentrations for both the experimental and calculated results. The N atom balance is calculated as the sum of nitrogen atoms contained in NH_3_, NO, NO_2_, N_2_O, and N_2_ divided by the inlet concentration of nitrogen atoms. As mentioned previously, the concentrations of NO and NO_2_ found in this work were always below the detection limit. Thus, the main contribution to the N atom balance in the experiments of the present work comes from NH_3_, N_2_, and N_2_O. Similarly, the C atom balance is calculated as the sum of carbon atoms contained in DMM, CO, and CO_2_ divided by the inlet concentration of carbon atoms. For N atoms, the balance closes above 96%, indicating that the nitrogen species measured in the experiments are clearly the dominant ones, in particular, NH_3_ and the formed N_2_ and N_2_O. In the case of C atoms, the balance reaches values lower than those of N atoms, and they are above 52%. This means that during the oxidation of NH_3_-DMM mixtures, other carbon species different from CO and CO_2_ are formed and that they have not been quantified due to not having the gas cylinder for equipment calibration. An analysis of the calculations from the model allowed one to identify that the probably missing carbon species are mainly formaldehyde and methyl formate, which are products detected in the high-pressure oxidation of neat DMM.^29^ The model also indicates that small amounts of hydrogen cyanide (HCN) and isocyanic acid (HNCO) may form. These compounds have been identified as important intermediate species in the combustion of mixtures of NH_3_ with hydrocarbons.^54,55^ Figure 6c,d presents the calculated N and C atom balances including (W) the model calculated concentrations of CH_2_O, CH_3_OCHO, HCN, and HNCO. A clear improvement is obtained in all the temperature ranges studied compared with the calculated N and C atom balances without including the model calculated concentrations of CH_2_O, CH_3_OCHO, HCN, and HNCO (Figure 6a,b), mainly for the C balance.

**Figure 6:**
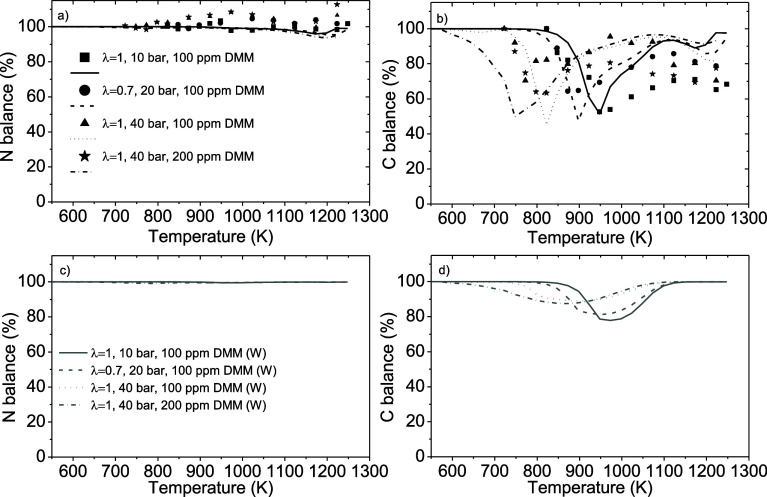
N and C balances during the oxidation of NH_3_-DMM mixtures, as a function of the temperature, for different stoichiometries, pressures, and inlet DMM concentrations. Sets 2, 4, 8, and 11 in Table 1. (a, b) Experimental and calculated balance not including the concentrations of CH_2_O, CH_3_OCHO, HCN, and HNCO. (c, d) Calculated balance including (W) the concentrations of CH_2_O, CH_3_OCHO, HCN, and HNCO.

The influence of the pressure on the oxidation of NH_3_-DMM mixtures has also been evaluated at different λ values (0.7, 1, and 3) and for inlet concentrations of ammonia and DMM of approximately 1000 and 100 ppm, respectively, and also for an inlet DMM concentration of around 200 ppm and λ = 1. Figure 7 shows, as an example, the concentration profile of reactants and products for λ = 0.7 and an inlet DMM concentration of 100 ppm. The concentration profiles of reactants and products for 100 ppm of DMM and λ = 1 and 3 and for 200 ppm of DMM and λ = 1 can be seen in Figures S6–S8, respectively, in the Supporting Information.

**Figure 7:**
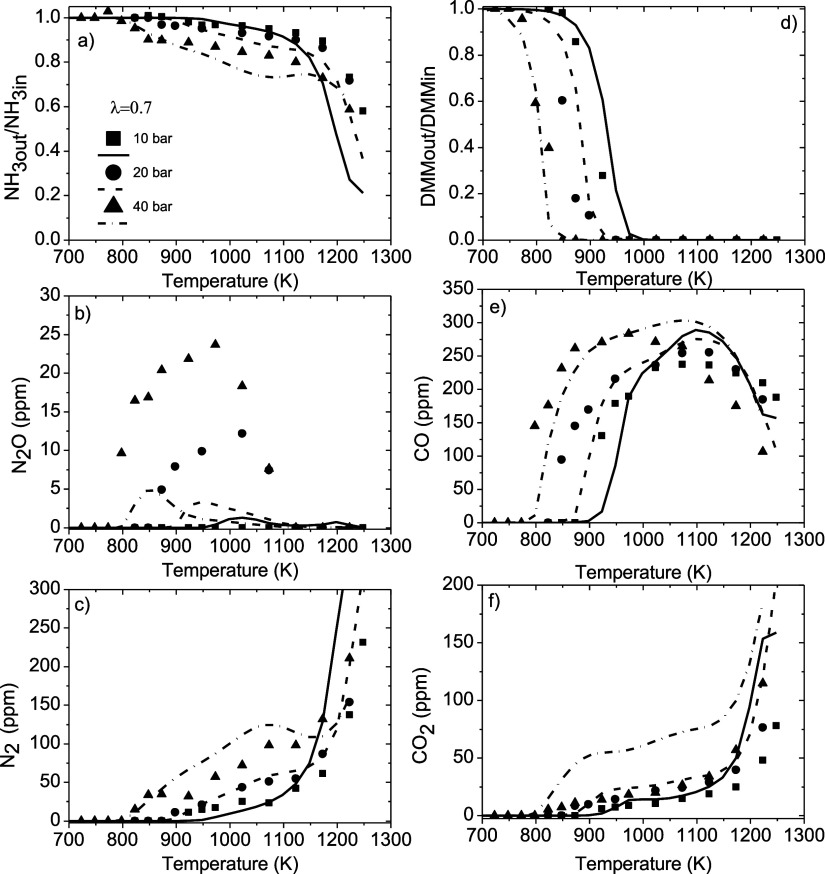
Influence of the pressure on the concentration profiles of (a) NH_3_, (b) N_2_O, (c) N_2_, (d) DMM, (e) CO, and (f) CO_2_ during the NH_3_-DMM mixture oxidation as a function of the temperature for λ = 0.7 and inlet DMM concentration of 100 ppm. Experimental results are denoted by symbols, and modeling calculations are denoted by lines. Sets 1, 4, and 7 in Table 1.

Figure 7 and Figure S6–S8 show that, for a given λ value, the experimental and calculated onset temperature for the conversion of ammonia and DMM is shifted to lower temperatures with the increase of pressure, which could be attributed to the increase in the absolute concentration of reactants (NH_3_, DMM, and O_2_). This behavior coincides with that observed in the high-pressure oxidation of neat ammonia^9^ and DMM.^29^ The increase in the amounts of oxygen and DMM in the reactant mixture emphasizes the shifts.

For any of the experimental conditions studied in the present work, the conversion of ammonia and DMM seems to start at the same temperature, but DMM is consumed in a shorter temperature interval than ammonia. For example, in the NH_3_-DMM mixture with 100 ppm of DMM with λ = 0.7 at 10 bar and 948 K (Figure 3), the conversion of DMM is 100% and that of ammonia is around 3%. A complete conversion of DMM is achieved in the temperature window studied for all of the experimental conditions, and it is observed that when DMM is totally consumed, the consumption of ammonia seems to increase. This could be attributed to the fact that the competition between ammonia and DMM for OH radicals (reaction 3 for ammonia and reactions 14 and 15 for DMM) no longer exists. However, a complete conversion of ammonia is not achieved in any of the experiments, with some amount still remaining at the maximum temperature studied (1250 K), even in the most beneficial conditions for its conversion (200 ppm of DMM, λ = 3, and 40 bar), where a conversion of around 95% is obtained. A reason for a nontotal conversion of ammonia could be because of the competition of ammonia, at high temperatures, for OH radicals with other DMM oxidation intermediates, as is the case of formaldehyde that reacts with OH to form HCO (reaction 8) and finally produces CO (reaction 9). Also, calculations indicate that the interaction of intermediates such as methyl formate and formaldehyde with NH_2_ radicals recycles back a small amount of these radicals into ammonia (reactions 4, 6, and 8), which has been called the NH_3_-NTC behavior,^56^ extending in this way the conversion regime of NH_3_ to a larger temperature interval compared to the oxidation of net ammonia. Nevertheless, DMM seems to promote the consumption of ammonia since in the work of García-Ruiz et al.^9^ on the high-pressure oxidation of approximately 1000 ppm of neat ammonia in the temperature range of 600–1275 K, using the same experimental facility and similar experimental conditions as in the present work, the temperature onset for the conversion of ammonia was above 1165 K, while in the presence of DMM, the highest temperature found in the present work for the onset of the conversion of ammonia was around 925 K (found for set 1 in Table 1). This result is in accordance with that observed by Elbaz et al.,^18^ who found that the addition of DMM enhanced the unstretched laminar burning velocity of ammonia significantly.

To better figure out the promotion of ammonia consumption in the presence of DMM, Figure 8 (λ = 1 at 40 bar) and Figures S9 (λ = 1 at 20 bar) and S10 (λ = 3 at 40 bar) show the effect of the inlet DMM concentration on the oxidation of NH_3_-DMM mixtures. Figure 8 also includes the experimental profiles for NH_3_, N_2_O, and N_2_ obtained by Garcia-Ruiz et al.^9^ on the high-pressure oxidation of neat NH_3_ at the same experimental conditions as in the NH_3_/DMM mixture, i.e., 40 bar and λ = 1.

**Figure 8:**
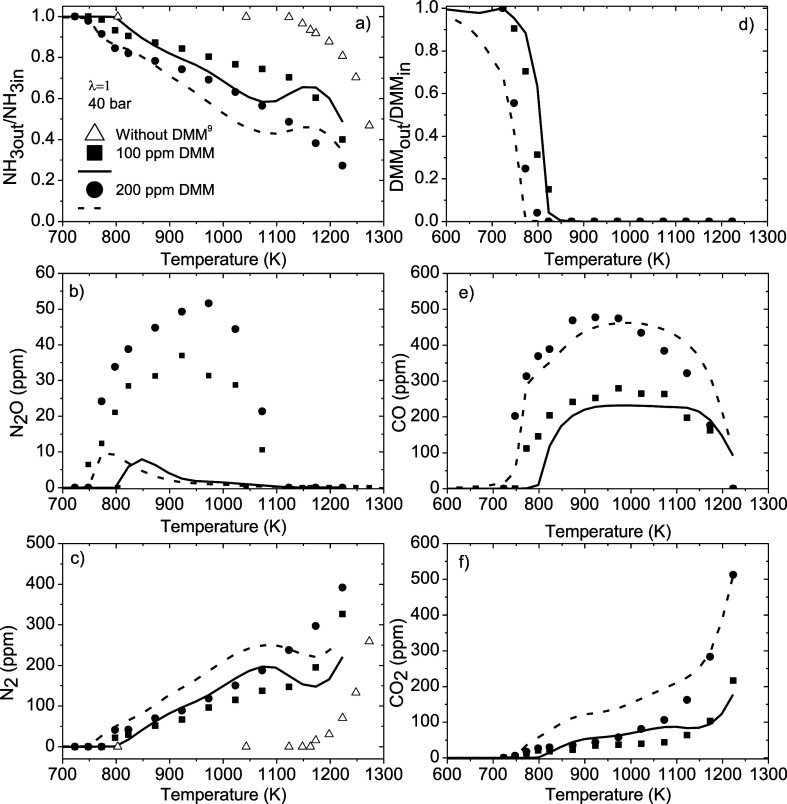
Influence of the inlet DMM concentration on the concentration profiles of (a) NH_3_, (b) N_2_O, (c) N_2_, (d) DMM, (e) CO, and (f) CO_2_ during the NH_3_–DMM mixture oxidation as a function of the temperature at 40 bar and λ = 1. Experimental results are denoted by symbols, and modeling calculations are denoted by lines. Sets 8 and 11 in Table 1 and profiles on the high-pressure oxidation of neat NH_3_ reproduced from García-Ruiz et al.^9^

It is observed that the presence of DMM shifts the start of NH_3_ consumption approximately 380 K less, and an increase in the inlet DMM concentration implies a shift to lower temperatures in the conversion temperature of both ammonia and DMM of approximately 25 K when the concentration is doubled. As mentioned above, the consumption of ammonia occurs through its reaction with OH radicals (reaction 3). As the building of the O/H radical pool is favored in the presence of DMM, there is more OH to react with ammonia than if there were no DMM. This is the reason for the importance of DMM to accelerate the consumption of ammonia. Thus, the higher the inlet DMM concentration is, the higher is the consumption of ammonia. However, it must be taken into account that the presence of DMM leads to the formation of N_2_O, and the higher the pressure and the concentration of O_2_ and DMM are, the greater is the amount of N_2_O formed (Figures 3, 7, and 8). Garcia-Ruiz et al.^25^ observed a similar outcome when studying the high-pressure oxidation of NH_3_-DME mixtures. N_2_O is a powerful greenhouse gas that, as mentioned above, can react with CO to produce CO_2_. This implies that the addition of oxygenated compounds to ammonia could represent a drawback in the control of this greenhouse gas.

## 4 Conclusions

A modeling and experimental study of the oxidation of NH_3_-DMM mixtures has been performed in a quartz tubular flow reactor at high pressure (10, 20, and 40 bar) and in the 650–1250 K temperature range. Different stoichiometries (λ = 0.7, 1, and 3) and inlet DMM concentrations (100 and 200 ppm) were considered. The experimental data were interpreted in terms of a detailed chemical–kinetic mechanism that was able to describe the main trends of the oxidation of NH_3_-DMM mixtures under the studied conditions.

Experimental and modeling results indicate that the presence of DMM promotes the conversion of NH_3_ and that the pressure and concentration of oxygen and DMM were key factors in the process. The higher the pressure and the levels of oxygen and DMM in the reaction environment are, the lower is the temperature onset for the conversion of ammonia and DMM. However, these conditions favor the formation of N_2_O, which reacts at high temperatures with CO to produce N_2_ and CO_2_. Neither NO nor NO_2_ was detected at any of the conditions of the present work.

The presence of DMM has a promoting role in the oxidation of ammonia reactivity. However, while a complete conversion of DMM is achieved in the temperature window studied, the same did not happen for ammonia. This could be because, at high temperatures, ammonia still competes with other DMM oxidation intermediates for OH radicals, and also some of these intermediates can react with the NH_2_ radicals to yield ammonia again_,_ which is recycled back to the reaction system.

## Supplementary Material







## Notes

Mechanism file (TXT)
Thermodynamic data (TXT)
Validation of the kinetic mechanism and figures of the influence of stoichiometry, pressure, and inlet DMM concentration on the concentration profile of species (DOCX)

## References

[1] Valera-Medina A., Amer-Hatem F., Azad A. K., Dedoussi I. C., de Joannon M., Fernandes R. X., Glarborg P., Hashemi H., He X., Mashruk S., McGowan J., Mounaim-Rouselle C., Ortiz-Prado A., Ortiz-Valera A., Rossetti I., Shu B., Yehia M., Xiao H., Costa M. (2021). Review on ammonia as
a potential Fuel: from synthesis to economics. Energy Fuels.

[2] Herbinet O., Bartocci P., Dana A. G. (2022). On the
use of ammonia as a fuel –
A perspective. Fuel Commun..

[3] Bardow A., Shter G. E., Grader N G.S., Grinberg D. A., Elishav O. (2016). Nitrogen-based
fuels: a power-to-fuel-to-power analysis. Angew.
Chem. Int. Ed.

[4] Gross C. W., Kong S. C. (2013). Performance characteristics
of a compression-ignition
engine using direct-injection ammonia–DME mixtures. Fuel.

[5] Mathieu O., Petersen E. L. (2015). Experimental and
modeling study on the high temperature
oxidation of ammonia and related NO_x_ chemistry. Combust. Flame.

[6] Song Y., Hashemi H., Christensen J. M., Zou C., Marshall P., Glarborg P. (2016). Ammonia oxidation at high pressure
and intermediate
temperatures. Fuel.

[7] Abián M., Benés M., de Goñi A., Muñoz B., Alzueta M. U. (2021). Study of the oxidation
of ammonia in a flow reactor&nbsp;Experiments
and kinetic modeling simulation. Fuel.

[8] Tang R., Xu Q., Pan J., Gao J., Wang Z., Wei H., Shu G. (2022). An experimental and
modeling study of ammonia oxidation in a jet
stirred reactor. Combust. Flame.

[9] García-Ruiz P., Uruén M., Abián M., Alzueta M. U. (2023). High pressure ammonia
oxidation in a flow reactor. Fuel.

[10] Herbinet O., Bartocci P., Dana A. G. (2022). On the
use of ammonia as a fuel –
A perspective. Fuel Comm..

[11] Pochet M., Dias V., Moreau B., Foucher F., Jeanmart H., Contino F. (2019). Experimental and numerical study,
under LTC conditions,
of ammonia ignition delay with and without hydrogen addition. Proc. Combust. Inst..

[12] Dai L., Gersen S., Glarborg P., Mokhov A., Levinsky H. (2020). Autoignition
studies of NH_3_/CH_4_ mixtures at high pressure. Combust. Flame.

[13] Li M., He X., Hashemi H., Glarborg P., Lowe V. M., Marshall P., Fernandes R., Shu B. (2022). An experimental and modeling study
on auto-ignition kinetics of ammonia/methanol mixtures at intermediate
temperature and high pressure. Combust. Flame.

[14] Li M., Zhu D., He X., Moshammer K., Fernandes R., Shu B. (2023). Experimental and kinetic
modeling study on auto-ignition properties
of ammonia/ethanol blends at intermediate temperatures and high pressures. Proc. Combust. Inst..

[15] Yin G., Li J., Zhou M., Li J., Wang C., Hu E., Huang Z. (2022). Experimental and kinetic study on laminar flame speeds
of ammonia/dimethyl
ether/air under high temperature and elevated pressure. Combust. Flame.

[16] Issayev G., Giri B. R., Elbaz A. M., Shrestha K. P., Mauss F., Roberts W. L., Farooq A. (2021). Combustion
behavior of ammonia blended
with diethyl ether. Proc. Combust. Inst..

[17] Dai L., Yuan Y., Lin Q., Li W., Zou C., Liu J., Luo J. (2023). Shock tube and modeling
study on the ignition delay
times of ammonia/dimethoxymethane at high temperature. Combust. Flame.

[18] Elbaz A. M., Giri B. R., Issayev G., Shrestha K. P., Mauss F., Farooq A., Roberts W. L. (2020). Experimental
and kinetic modeling
study of laminar flame speed of dimethoxymethane and ammonia blends. Energy Fuels.

[19] Wang S., Wang Z., Elbaz A. M., Han X., He Y., Costa M., Konnov A. A., Roberts W. L. (2020). Experimental
study
and kinetic analysis of the laminar burning velocity of NH_3_/syngas/air, NH_3_/CO/air and NH_3_/H_2_/air premixed flames at elevated pressures. Combust. Flame.

[20] Chen Z., Jiang Y. (2021). Numerical investigation
of the effects of H_2_/CO/syngas
additions on laminar premixed combustion characteristics of NH_3_/air flame. Int. J. Hydrogen Energy.

[21] Zhu S., Xu Q., Tang R., Gao J., Wang Z., Pan J., Zhang D. (2023). A comparative study
of oxidation of pure ammonia and ammonia/dimethyl
ether mixtures in a jet-stirred reactor using SVUV-PIMS. Combust. Flame.

[22] Alexandrino K., Millera Á., Bilbao R., Alzueta M. U. (2018). Gas and soot formed
in the dimethoxymethane pyrolysis. Soot characterization. Fuel Process. Technol..

[23] Dai L., Hashemi H., Glarborg P., Gersen S., Marshall P., Mokhov A., Levinsky H. (2021). Ignition delay
times of NH_3_/DME blends at high pressure and low DME fraction:
RCM experiments
and simulations. Combust. Flame.

[24] Xiao H., Li H. (2022). Experimental and kinetic
modeling study of the laminar burning velocity
of NH_3_/DME/air premixed flames. Combust.
Flame.

[25] García-Ruiz P., Ferrando P., Abián M., Alzueta M. U. (2025). High-pressure conversion
of ammonia additivated with dimethyl ether in a flow reactor. Combust. Flame.

[26] Zhang C., Li P., Li Y., He J., Li X. (2014). Shock-tube study of
dimethoxymethane ignition at high temperatures. Energy Fuels.

[27] Li R., Herreros J. M., Tsolakis A., Yang W. (2021). Chemical kinetic modeling
of diethoxymethane oxidation: a carbon-neutral fuel. Fuel.

[28] Zhang X., Oyedun A. O., Kumar A., Oestreich D., Arnold U., Sauer J. (2016). An optimized process design for oxymethylene
ether production from woody-biomass-derived syngas. Biomass and Bioenergy.

[29] Marrodán L., Royo E., Millera A., Bilbao R., Alzueta M. U. (2015). High pressure
oxidation of dimethoxymethane. Energy Fuels.

[30] Marrodán L., Monge F., Millera A., Bilbao R., Alzueta M. U. (2016). Dimethoxymethane
oxidation in a flow reactor. Combust. Sci. Technol..

[31] Sun W., Tao T., Lailliau M., Hansen N., Yang B., Dagaut P. (2018). Exploration
of the oxidation chemistry of dimethoxymethane: jet-stirred reactor
experiments and kinetic modeling. Combust. Flame.

[32] Zhang Y., Wang Q., Dai L., Zhang M., Yu C. (2023). Numerical
study on the combustion properties of ammonia/DME and ammonia/DMM
mixtures. Energies.

[33] Marrodán L., Millera A., Bilbao R., Alzueta M. U. (2014). High-pressure study
of methyl formate oxidation and its interaction with NO. Energy Fuels.

[34] García-Ruiz P., Salas I., Casanova E., Bilbao R., Alzueta M. U. (2024). Experimental
and modeling high-pressure study of ammonia–methane oxidation
in a flow reactor. Energy Fuels.

[35] Glarborg P., Miller J. A., Ruscic B., Klippenstein S. J. (2018). Modeling
nitrogen chemistry in combustion. Prog. Energy
Combust..

[36] Stagni A., Cavallotti C., Arunthanayothin S., Song Y., Herbinet O., Battin-Leclerc F., Faravelli T. (2020). An experimental, theoretical and
kinetic-modeling study of the gas-phase oxidation of ammonia. React. Chem. Eng..

[37] Alzueta M. U., Guerrero M., Millera A., Marshall P., Glarborg P. (2021). Experimental
and kinetic modeling study of oxidation of acetonitrile. Proc. Comb. Inst..

[38] Marrodán L., Pérez T., Alzueta M. U. (2024). Conversion of methylamine
in a flow
reactor and its interaction with NO. Combust.
Flame.

[39] Burke M. P., Klippenstein S. J. (2017). Ephemeral
collision complexes mediate chemically termomolecular
transformations that affect system chemistry. Nat. Chem..

[40] Klippenstein S. J., Sivaramakrishnan R., Burke U., Somers K. P., Curran H. J., Cai L., Pitsch H., Pelucchhi M., Faravelli T., Glarborg P. (2022). HÉ2+HÉ2: High level theory and the role
of singlet channels. Combust. Flame.

[41] Marshall P., Rawling G., Glarborg P. (2021). New reactions
of diazene and related
species for modelling combustion of amine fuels. Mol. Phys..

[42] Alzueta M. U., Ara L., Mercader V. D., Delogu M., Bilbao R. (2022). Interaction of NH3
and NO under combustion conditions. Experimental flow reactor study
and kinetic modeling simulation. Combust. Flame.

[43] Alzueta M. U., Ara L., Mercader V. D., Delogu M., Bilbao R. (2022). Interaction of NH3
and NO under combustion conditions. Experimental flow reactor study
and kinetic modeling simulation. Combust. Flame.

[44] Glarborg P., Hashemi H., Cheskis S., Jasper A. W. (2021). On the rate constant
for NH2+HO2 and third-body collision efficiencies for NH_2_ + H­(+M) and NH_2_ + NH_2_ (+M). J. Phys. Chem. A.

[45] Glarborg P., Hashemi H., Marshall P. (2022). Challenges in kinetic
modeling of
ammonia pyrolysis. Fuel Comm..

[46] Glarborg P. (2023). The NH_3_/NO_2_/O_2_ system: Constraining key steps
in ammonia ignition and N_2_O formation. Combust. Flame.

[47] Marrodán L., Millera A., Bilbao R., Alzueta M. U. (2022). Experimental and
modeling evaluation of dimethoxymethane as an additive for high-pressure
acetylene oxidation. J. Phys. Chem. A.

[48] Vermeire F. H., Carstensen H. H., Herbinet O., Battin-Leclerc F., Marin G. B., Van Geem K. (2018). Experimental
and modeling study of
the pyrolysis and combustion of dimethoxymethane. Combust. Flame.

[49] Jacobs S., Döntgen M., Alquaity A. B. S., Kopp W. A., Kröger L. C., Burke U., Pitsch H., Leonhard K., Curran H. J., Heufer K. A. (2019). Detailed kinetic modeling of dimethoxymethane&nbsp;Part.
II: experimental and theoretical study of the kinetics and reaction
mechanism. Combust. Flame.

[50] Allen, J. W. Predictive chemical kinetics: enabling automatic mechanism generation and evaluation [PhD Thesis] 2013, Massachusetts Institute of Technology: Boston

[51] ANSYS ANSYS Chemkin 18: Chemical Kinetic Simulation software. Chemkin ANSYS Reaction Design. v. 18.2, San Diego, 2016 Date of access 5th may 2025. https://www.ansys.com/products/fluids/ansys-chemkin-pro.

[52] Alzueta M. U., Pérez T., Marrodán L. (2024). Oxidation of methylkamine CH3NH2/CH4/NO
mixtures in an atmospheric-pressure flow reactor. Proc. Comb. Inst..

[53] Marrodán L., Millera A., Bilbao R., Alzueta M. U. (2014). High-pressure
study
of methyl formate oxidation and its interaction with NO. Energy Fuels.

[54] Alzueta M. U., Salas I. (2024). Conversion of NH3/CO/NO/CO2 mixtures. Fuel.

[55] Li Y., Sarathy S. M. (2020). Probing hydrogen-nitrogen
chemistry: A theoretical
study of important reactions in N_x_H_y_, HCN and
HNCO oxidation. Int. J. Hydrogen Energy.

[56] García-Ruiz P., Ferrando P., Abián M., Alzueta M. U. (2025). High pressure conversión
of ammonia additivated with dimethyl ether in a flow reactor. Combust. Flame.

