# Supplementary material for: High-Pressure Oxidation of Ammonia Mixed with Dimethoxymethane
Source: Energy Fuels. 2025 Jul 9;39(29):14213–22. doi: 10.1021/acs.energyfuels.5c01020 (PMC12308821; doi:10.1021/acs.energyfuels.5c01020)
Supplement: Supplementary file 3 [file ef5c01020_si_003.docx]

Supplementary Material

High pressure oxidation of ammonia mixed with dimethoxymethane.

Katiuska Alexandrino, Álvaro Andrés, Alicia Callejas, María U. Alzueta*

Aragón Institute of Engineering Research (I3A), Department of Chemical and Environmental Engineering, University of Zaragoza, 50018 Zaragoza, Spain

*Corresponding author: e-mail address: [uxue@unizar.es](mailto:uxue@unizar.es)

Figure. S1. Temperature profiles at 40 bar for different temperatures.

Figure S2. Ignition delay time measurements for NH_3_/DMM/O_2_/Ar mixtures obtained by Dai et al.^17^ and the simulation results obtained with the model proposed in the present work.

Figure S3. Influence of the stoichiometry on the concentration profiles of: a) NH_3_, b) N_2_O, c) N_2_, d) DMM, e) CO and f) CO_2_ during the NH_3_−DMM mixture oxidation as a function of the temperature at 20 bar and an inlet DMM concentration of 100 ppm. Experimental results are denoted by symbols, and modeling calculations are denoted by lines. Sets 4- 6 in Table 1.

Figure S4. Influence of the stoichiometry on the concentration profiles of: a) NH_3_, b) N_2_O, c) N_2_, d) DMM, e) CO and f) CO_2_ during the NH_3_−DMM mixture oxidation as a function of the temperature at 40 bar and an inlet DMM concentration of 100 ppm. Experimental results are denoted by symbols, and modeling calculations are denoted by lines. Sets 7- 9, and R9 in Table 1.

Figure S5. Influence of the stoichiometry on the concentration profiles of: a) NH_3_, b) N_2_O, c) N_2_, d) DMM, e) CO and f) CO_2_ during the NH_3_−DMM mixture oxidation as a function of the temperature at 40 bar and an inlet DMM concentration of 200 ppm. Experimental results are denoted by symbols, and modeling calculations are denoted by lines. Sets 11 and 12 in Table 1.

Figure S6. Influence of the pressure on the concentration profiles of: a) NH_3_, b) N_2_O, c) N_2_, d) DMM, e) CO and f) CO_2_ during the NH_3_−DMM mixture oxidation as a function of the temperature for λ=1 and inlet DMM concentration of 100 ppm. Experimental results are denoted by symbols, and modeling calculations are denoted by lines. Sets 2, 5 and 8 in Table 1.

Figure S7. Influence of the pressure on the concentration profiles of: a) NH_3_, b) N_2_O, c) N_2_, d) DMM, e) CO and f) CO_2_ during the NH_3_−DMM mixture oxidation as a function of the temperature for λ=3 and inlet DMM concentration of 100 ppm. Experimental results are denoted by symbols, and modeling calculations are denoted by lines. Sets 3, 6 and 9 in Table 1.

Figure S8. Influence of the pressure on the concentration profiles of: a) NH_3_, b) N_2_O, c) N_2_, d) DMM, e) CO and f) CO_2_ during the NH_3_−DMM mixture oxidation as a function of the temperature for λ=1 and inlet DMM concentration of 200 ppm. Experimental results are denoted by symbols, and modeling calculations are denoted by lines. Sets 10 and 11 in Table 1.

Figure S9. Influence of the inlet DMM concentration on the concentration profiles of: a) NH_3_, b) N_2_O, c) N_2_, d) DMM, e) CO and f) CO_2_ during the NH_3_−DMM mixture oxidation as a function of the temperature at 20 bar and λ=1. Experimental results are denoted by symbols, and modeling calculations are denoted by lines. Sets 5 and 10 in Table 1.

Figure S10. Influence of the inlet DMM concentration on the concentration profiles of: a) NH_3_, b) N_2_O, c) N_2_, d) DMM, e) CO and f) CO_2_ during the NH_3_−DMM mixture oxidation as a function of the temperature at 40 bar and λ=3. Experimental results are denoted by symbols, and modeling calculations are denoted by lines. Sets 9 and 12 in Table

1.
